# Effect of Pulpal Base, Restorative Material, and Preparation Type on Marginal and Internal Fit and Fracture Strength of Endocrowns

**DOI:** 10.3390/ma18092137

**Published:** 2025-05-06

**Authors:** Kerem Yılmaz, Hakan Aydın, Fehmi Gönüldaş, Sukan Kara, Özge Çiloğlu, Erdem Özdemir, Zeynep Bilen

**Affiliations:** 1 Department of Prosthodontics, Faculty of Dentistry, Antalya Bilim University, Bilimdent Oral and Dental Health Center, Antalya 07040, Turkey; 2 Department of Endodontics, Faculty of Dentistry, Antalya Bilim University, Bilimdent Oral and Dental Health Center, Antalya 07040, Turkey; 3 Department of Prosthodontics, Faculty of Dentistry, Ankara University, Ankara 06560, Turkey; fgonuldas@ankara.edu.tr (F.G.);; 4 Department of Oral and Maxillofacial Radiology, Faculty of Dentistry, Antalya Bilim University, Bilimdent Oral and Dental Health Center, Antalya 07040, Turkey; 5 Private Practice, Özdemir Dental Center, Antalya 07160, Turkey

**Keywords:** endocrown, hybrid ceramic, fiber ribbon, computer-aided design, tooth preparation, marginal fit, triple scan, fracture strength

## Abstract

The aim of this innovative study was to investigate the feasibility of a modified butt joint preparation with two grooves instead of a ferrule when root dentin tissue is limited in mandibular molars. It was also investigated to what extent the effect of these treatment options on marginal and internal fit and fracture strength (FS) varied according to the type of material and whether or not a fiber ribbon was used at the base. Marginal and internal fit were evaluated using the triple-scan protocol. Statistical analysis was conducted via a three-way analysis of variance (ANOVA). The absolute marginal discrepancy (AMD), marginal discrepancy, and overall fit values for the ceramic group were 127 μm, 108 μm, and 120 μm, respectively, while corresponding values for the hybrid ceramic group were 139 μm, 116 μm, and 130 μm (*p* < 0.05). The mean FS recorded for ceramic restorations was 662 N, whereas hybrid ceramic restorations demonstrated a significantly higher FS of 903 N (*p* < 0.001). When material type was assessed independently of preparation design and base configuration, both ceramic and hybrid ceramic exceeded the predefined clinical acceptability threshold for AMD; however, they remained within acceptable limits for the remaining parameters. Among the evaluated configurations, hybrid ceramic restorations incorporating ferrules and fiber-reinforced bases exhibited the highest FS values, whereas ceramic restorations with modified biological widths and lacking fiber reinforcement yielded the lowest FS values.

## 1. Introduction

Restoring severely damaged endodontically treated teeth presents biomechanical challenges [1]. Advances in adhesive technology have introduced minimally invasive treatments, and in 1999, a type of restoration called “endocrown” was introduced that does not contain an intra-canal retainer. The restoration was placed in a cavity prepared in the center of a tooth. The preparation margin had a flat shape called the “butt margin”. After two years of use, this type of restoration was found to be clinically acceptable [2].

Fracture strength (FS) represents a key parameter in evaluating the biomechanical integrity and clinical durability of dental restorations. The type of material and preparation and the marginal and internal fit are among the factors that influence the FS of endocrowns [3]. Various preparation designs exist, primarily distinguished by the type of finish line and the configuration of the pulpal cavity. Two main finish line types are recognized: the butt joint (BJ) and the ferrule. The BJ design features a 1–2 mm wide flat enamel band oriented at a 90° angle to the axial wall. In contrast, the ferrule design incorporates a shoulder or chamfer finish line that also forms a 90° angle, but includes additional axial wall height above the enamel band. Although limited in number, existing studies investigating the impact of finish line configuration on FS have yielded mixed results, some indicating a superior performance for the BJ design, while others favor the ferrule [4,5]. A few investigations have explored modified finish line configurations. For instance, one study employing a BJ design varied the vertical positioning of the finish line relative to the cementoenamel junction (CEJ) [6], whereas another study introduced axial walls of differing depths within the same preparation [7]. These findings underscore the need for further research to clarify the influence of finish line geometry on the FS of restorations.

Numerous studies have investigated the effect of pulp chamber design on the FS of endocrowns. In one study [8], low FS was reported in all endocrowns regardless of pulp chamber depth. Another study [9] reported higher FS in deep endocrowns compared to shallow pulp chambers. Several studies [10,11] have reported that deep indentations at the base of the pulp chamber increase FS [12,13] and that the remaining tooth tissue or normal anatomy of the pulp chamber walls should be preserved.

The production of endocrowns that provide a long-term high seal is dependent on the structures of both the sealing materials used in the root canal treatment and the base materials used in the preparation of the pulp cavity [14]. Resin-containing materials are commonly employed as pulp base materials [10]. There is no consensus about incorporating fiber into the pulp base, as studies have found contradictory findings concerning the possibility of crack propagation modification from irreparable to repairable [15], or no significant effect on either FS or crack pattern [16]. These varying outcomes highlight the necessity for comprehensive investigations that incorporate modified preparation designs to better elucidate the role of base materials in restorative performance.

On the other hand, studies that examined the effect of marginal or internal fit on FS focused on the finish line, restorative material, or pulpal design [11,17,18]. In some previous studies, the marginal fit was analyzed using two reference points called marginal discrepancy (MD) and absolute marginal discrepancy (AMD) [19,20]. Holmes et al. [21] defined the MD as the perpendicular measurement from the cervical edge of the crown to the edge of the preparation and the AMD as the angular combination of the MD and the extension error (over-extension or under-extension). An increase in MD leads to an increase in microleakage, and consequently an increase in the amount of cement material exposed to the oral environment and dissolved. AMD is an indicator of indentation or protrusion of the crown margin relative to the finish line and is important because it increases plaque accumulation [22]. MD and AMD parameters were used in the few studies in which the marginal and internal fit of computer-aided design/computer-aided manufacturing (CAD/CAM)-fabricated restorations were evaluated by triple scan.

For these reasons, the aim of this study was to investigate the effect of restorative material, preparation, and base material type on FS and marginal and internal fit in endocrowns. The first null hypothesis was that material, preparation, and base type would not affect marginal and internal fit, and the second null hypothesis was that they would not affect FS.

## 2. Materials and Methods

Ethical approval was obtained from the Antalya Bilim University Health Sciences Non-Interventional Research Ethics Committee prior to the start of the study (2024/033). The process was divided into six stages, as follows: (1) tooth selection and endodontic treatment, (2) base and tooth preparation, (3) restoration production and fit measurements, (4) permanent cementation, (5) thermomechanical aging and FS testing, and (6) stereomicroscopic assessment. For standardization and randomization, each stage was performed by a different and single person.

The sample size was calculated using a power analysis software (G*Power version 3.1.9.7; Heinrich Heine University, Düsseldorf, Germany). Based on a significance level (α) of 0.05, a statistical power (1−β) of 0.80, and an expected effect size (f) of 0.40, the minimum required sample size was determined to be 52 [23].

Mandibular first molars newly extracted for periodontal reasons were collected for this study. The crown and root dimensions of the teeth were measured using a digital caliper (Caliper; Yamer Industry, İzmir, Turkey), and teeth with similar dimensions were included in the study. Other inclusion criteria were absence of caries, absence of visible cracks, absence of restoration or root canal treatment, and complete root formation. The teeth were stored in 0.1% thymol solution at 4 °C for disinfection immediately after extraction until the start of the study. The storage time in the solution was set to a maximum of 1 week in order to maintain natural conditions. The materials used in the study are listed in Table 1.

**Figure 1:**
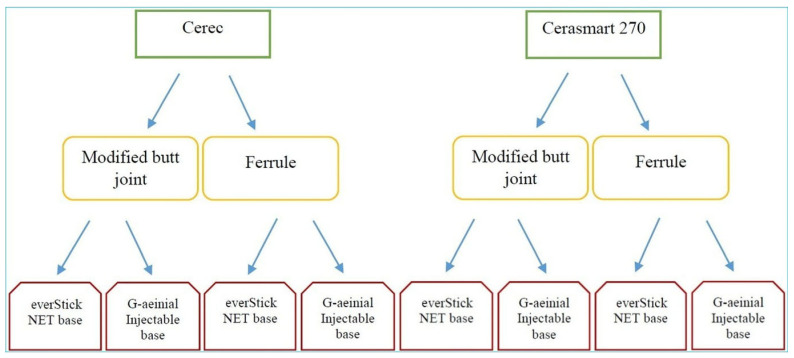
Group design.

Two main groups were formed according to the type of restorative material (*n* = 32). The groups were divided into two groups according to the type of preparation (*n* = 16). These groups were also divided into two groups according to the type of base (*n* = 8). Teeth were randomly selected for the groups (Figure 1).

Teeth were cut horizontally 2 mm above the CEJ using a water-cooled separator (IsoMet 5000; Buehler, IL, USA). Root canals were prepared using a 10 K file (K-file; Dentsply-Maillefer, Ballaigues, Switzerland). Chemomechanical root canal preparation was performed with a rotating file system (Protaper; Dentsply-Maillefer, Ballaigues, Switzerland). A 5.25% sodium hypochlorite solution (Calasept; Nordiska Dental, Angelholm, Sweden) was used for irrigation. The canals were filled with gutta-percha (Protaper Next; Sirona Dental, Behnsheim, Germany) using the lateral condensation method.

All tooth preparations were performed with the same set of diamond burs (Dental Burs; B&D Technologies, Guangzhou, China). Depth and width measurements were taken with a periodontal probe to ensure standardization.

The axial walls of the pulp chamber were refined using a round-end cylinder diamond bur (1.4 mm diameter, 6.0 mm length), while the axial bevel was standardized to an 8–10° taper using a double-cone diamond bur (3.5 mm diameter, 6.0 mm length) [24]. The preparation depth was set at 3.6 mm.

The pulp chamber walls were etched with 37% phosphoric acid (Ultra-etch; Ultradent, UT, USA). After a waiting period of 15 s, they were washed and air-dried, and a bonding agent (G-Premio Bond; GC Dental, Tokyo, Japan) was applied. After a waiting period of 10 s, they were cured for 20 s with an LED light source (Elipar S10; 3M ESPE, Seefeld, Germany).

**Figure 2:**
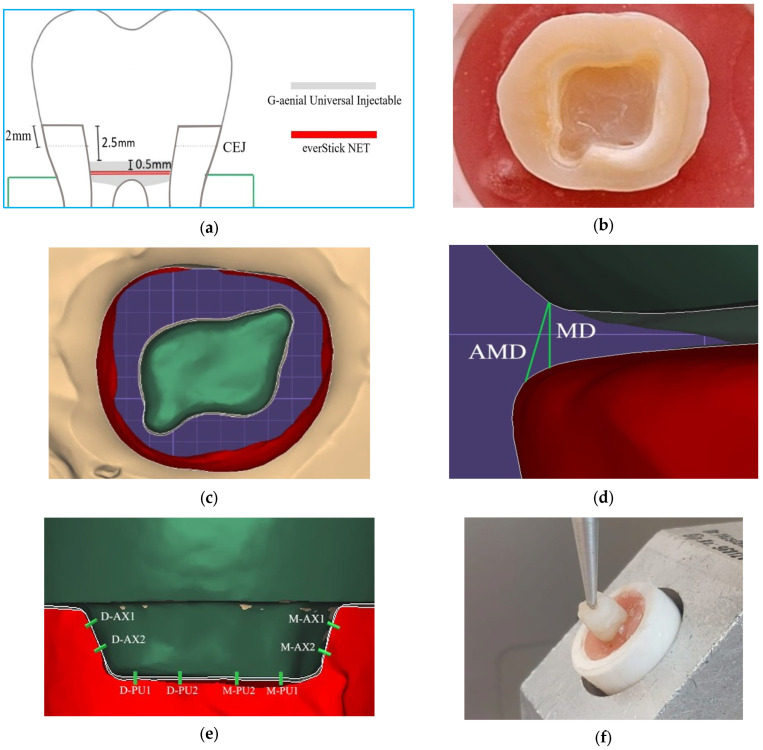
Endocrown preparation and testing process. (**a**) Design of base with fiber. CEJ, cementoenamel junction. (**b**) Cavity with modified butt joint. Final view of the preparation. (**c**) Horizontal section image obtained from the software program. (**d**) Marginal fit reference points. AMD, absolute marginal discrepancy. MD, marginal discrepancy. (**e**) Internal fit reference points in the mesiodistal section. D, distal; AX, axial; PU, pulpal; M, mesial. (**f**) Fracture strength test.

**Figure 3:**
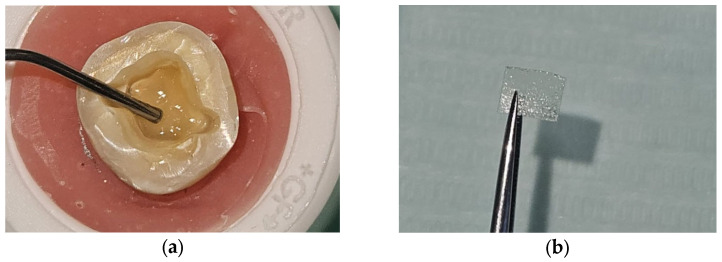

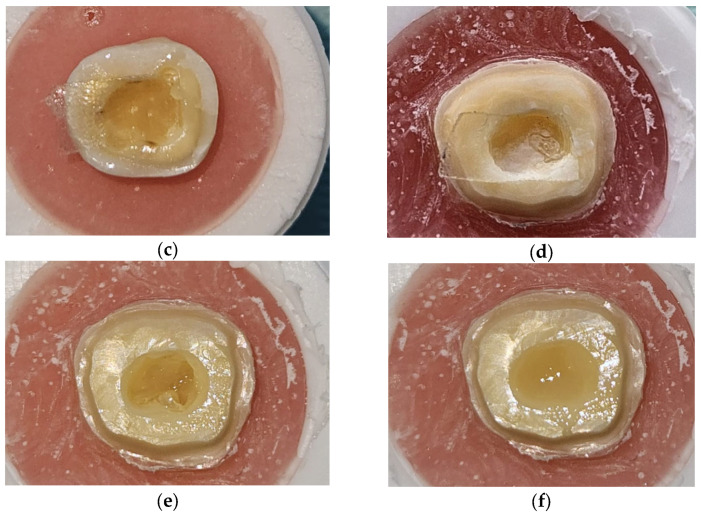
Fiber base preparation. (**a**) Application of flowable resin. (**b**) Fiber ribbon sectioning and recontouring. (**c**,**d**) Insertion of two layers of fiber ribbon into the cavity. (**e**,**f**) Additional flowable resin insertion to encapsulate the fibers.

In the group without fiber ribbon, a flowable resin (G-aenial Universal Injectable; GC Corp, Tokyo, Japan) was applied to the base and cured for 20 s. In the group with fiber ribbon, a thin layer of flowable resin (G-aenial Universal Injectable; GC Corp, Tokyo Japan) was applied to the cavity (0.5 mm thick), the cut and sized fiber ribbon was inserted into the resin in two layers (0.1 mm thick), and a second layer of flowable resin (0.5 mm thick) (G-aenial Universal Injectable; GC Corp, Tokyo, Japan) was applied on top of the fiber ribbon and cured for 20 s. The design and preparation of the base with fiber ribbon are shown in Figure 2a and Figure 3. The teeth were kept in distilled water at 4 °C for one day for standardization.

In this study, based on the results of studies comparing pulp cavity depths of 2.5 mm and 5 mm [3], 2 mm and 4 mm [8], and 1 mm and 5 mm [10], the distance from the tooth margin to the pulp base was set at 2.5 mm.

The enamel band was prepared to a minimum width of 2 mm. Retention grooves of 1.0 mm in depth and 2.5 mm in length parallel to the long axis of the tooth were made at the mesiobuccal and distolingual corners using a cylindrical diamond bur (1.2 mm diameter and 8 mm length) with a round end. The final tooth preparation with the modified BJ is shown in Figure 2b,c. The finish line of the ferrule had two differences: Firstly, no retention grooves were created in the pulpal region. Secondly, a ferrule design was created on the enamel band. A flat end cylinder diamond bur (1 mm diameter and 4 mm length) was used for this purpose. The ferrule was placed as a 2 mm high and 1 mm wide shoulder. The prepared teeth were immersed in distilled water at 4 °C for one day for standardization.

The restorative materials used in this study were manufactured using a CAD/CAM system. One was feldspathic (Cerec Blocs; Sirona Dental, Beinsheim, Germany [CE]) and the other was hybrid ceramic (Cerasmart 270; GC Corp, Tokyo, Japan [CS]). The materials used and the mechanical testing procedures applied in this study were in accordance with the reported EN ISO 6872:2024 standard for dental ceramic restorations.

The prepared teeth were scanned using an intraoral scanner (Cerec Omnicam 4.5; Sirona Dental, Bensheim, Germany). The “Comply with instrument geometry” button was disabled. The “Distance” button was used to standardize the cervicoocclusal lengths to 7 mm [25]. To avoid differences between the groups, the pulpal and axial cement spacing was set to 80 µm. The crowns were fabricated on a milling machine (Cerec MC XL; Sirona Dental, Bensheim, Germany) and polished with an appropriate polishing kit (EVE Flexi-Dia Ra; EVE Ernst Vetter, Keltern, Germany).

The triple scan was performed using the same intraoral scanner. Teeth were scanned in the “Lower jaw” tab, endocrowns in the “Upper jaw” tab, and teeth with endocrowns in the “Buccal” tab. The buccal tab with a green tick was moved to the “Biocopy sub” tab. The scan files generated in STL format were transferred to a CAD software (Exocad DentalCAD 3.1; Exocad, Darmstadt, Germany) to allow marginal and internal fit assessment.

For standardization, the teeth were kept in distilled water at 4 °C for 1 day and then permanent cementation was started. The inner surfaces of the restorations were etched with 5% hydrofluoric acid (IPS Ceramic Etching Gel; Ivoclar Vivadent, Schaan, Liechtenstein) for 60 s, and then silane (Ceramic Primer II; GC Corp, Tokyo, Japan) was applied. The teeth were etched with 37% phosphoric acid (Ultra-etch; Ultradent, UT, USA) for 15 s. A bonding agent (G-Premio Bond; GC Corp, Tokyo, Japan) was applied and cured for 20 s with an LED light source (Elipar S10; 3M ESPE, Seefeld, Germany). The restorations were cured with an adhesive resin (G-CEM One; GC Corp, Tokyo, Japan) under finger pressure for 60 s using the same light source [26]. After a curing time of 20 s on each surface, the teeth were kept in distilled water at 4 °C until thermomechanical aging was performed.

The triple-scan files were opened in the software (Exocad DentalCAD; Exocad, Darmstadt, Germany) in the following order: lower, upper, and buccal. The mesiodistal and buccolingual sections of the teeth were visualized using the “Regional view” button. The mesiodistal and buccolingual sections were determined using the center of the central fossa of the teeth as a guide.

Marginal and internal fit measurements were performed using the “Distance” tab. It was reported that measurement accuracy increased as the number of measurement points increased. A total of 12 measurements were taken in the buccolingual section, including AMD, MD, axial, and pulpal. The same 12 measurements were also taken in the mesiodistal section [27]. The reference points for marginal and internal fit are shown in Figure 2d,e.

Thermomechanical cyclic loading was performed using a thermocycler (SD Mechatronik Thermocycler; Julabo, Seelbach, Germany) with the ISO TR 11450:1999 standard. The specimens were subjected to 10,000 cycles in 5 °C and 55 °C water with a dwell time of 30 s to mimic 1 year of wear [28]. After thermomechanical aging, the specimens were stored in distilled water at 4 °C until fracture testing was performed.

The FS test was performed in accordance with the standard ISO 7500-1:1999. A load application angle of 45° was chosen to mimic a scenario where the force was concentrated in the cervical region [29]. The tip of the fracture tester (Lloyd Lrx; Lloyd Instruments, Bognor Regis, UK) with a 5 mm diameter steel ball was placed at an angle of 45° at the level of the central fossa of the teeth, at the beginning of the occlusolingual slopes of the buccal tubercles (Figure 2f) [25]. The fracture test started with a force of 50 N at a crosshead speed of 1.0 mm/min. The FS was recorded in Newtons (N).

Specimens were examined under a stereomicroscope (Leica MZ12; Leica Microsystems, Heerbrugg, Switzerland) at 8× magnification. Failure types were categorized as follows: Type I; cohesive failure in endocrown; Type II; adhesive failure between endocrown and dentin; Type III; cohesive failure in enamel/dentin; Type IV; fracture extending towards the root, above the CEJ; Type V; fracture extending below the CEJ (irreparable) [30].

Statistical analysis was performed using a software package (SPSS Statistics 30.0; IBM Corp, NY, USA). Descriptive statistics, including mean and standard deviation, were calculated for each group. The Kolmogorov–Smirnov test was used to test whether the distribution of each variable was normally distributed. Three-way analysis of variance (ANOVA) was performed to assess the interaction between the three independent variables—material, preparation, and base—and the main effects of each variable on marginal and internal fit and FS. Statistical significance was set at *p* < 0.05.

## 3. Results

According to the ANOVA results for marginal and internal fit, the effect of the material variable on AMD, MD, axial, pulpal, and overall fit was statistically significant (*p* < 0.05) (Table 2).

When the material was analyzed regardless of preparation and base, the AMD, MD, axial fit, pulpal fit, and overall fit values obtained for CE were 127 ± 23 µm, 108 ± 9 µm, 106 ± 9 µm, 139 ± 29 µm, and 120 ± 6 µm, respectively, while for CS they were 139 ± 23 µm, 116 ± 13 µm, 112 ± 10 µm, 151 ± 20 µm, and 130 ± 11 µm (*p* < 0.05) (Table 3).

When the preparation was analyzed regardless of material and base, the AMD, MD, axial fit, pulpal fit, and overall fit values obtained for the modified BJ were 135 ± 20 µm, 113 ± 9 µm, 107 ± 9 µm, 141 ± 25 µm, and 124 ± 10 µm, respectively, while for ferrule they were 131 ± 27 µm, 112 ± 13 µm, 111 ± 10 µm, 149 ± 26 µm, and 126 ± 10 µm (*p* > 0.05).

When the base was analyzed regardless of preparation and material, the AMD, MD, axial fit, pulpal fit, and overall fit values obtained for ES were 133 ± 23 µm, 111 ± 11 µm, 110 ± 11 µm, 152 ± 23 µm, and 126 ± 9 µm, respectively, while for GU they were 134 ± 25 µm, 113 ± 12 µm, 109 ± 9 µm, 138 ± 27 µm, and 123 ± 11 µm (*p* > 0.05).

**Figure 4:**
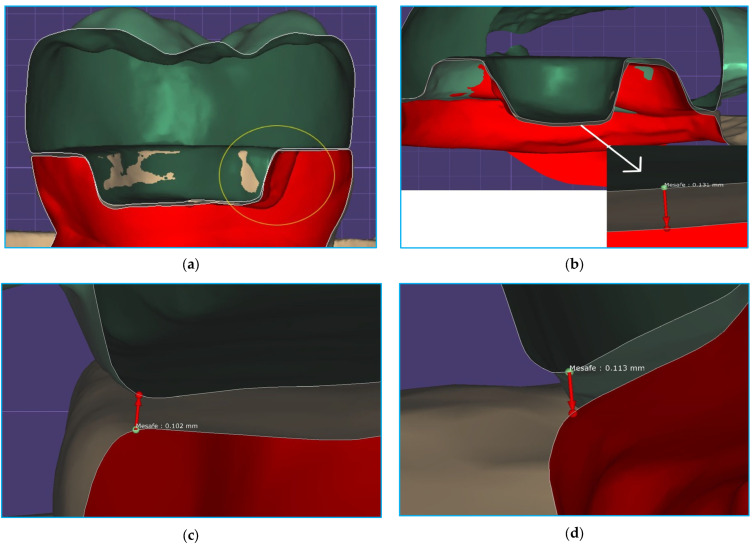
Fit assessment with computer software. (**a**) Mesiodistal section of a tooth with a modified butt joint. One of the retention grooves is seen in the yellow circle. (**b**) Buccolingual cross-sectional view of one of the hybrid ceramic restorations with ferrule and fiber. The measurement value from the lingual pulpal-2 reference point was 131 µm. (**c**) Marginal discrepancy measurement in restoration with fiber and ceramic. The value obtained was 102 µm. (**d**) Marginal discrepancy measurement in restoration without fiber and with hybrid ceramic. The value obtained was 113 µm.

Figure 4 shows the mesiodistal section in the tooth with the modified BJ; the buccolingual section in the tooth with ferrule, fiber ribbon, and CS; the mesial MD measurement in the tooth with ferrule, fiber ribbon, and CE; and the buccal MD measurement in the tooth with ferrule, without a fiber base, and with CS.

According to the ANOVA results of the FS, the effect of material, preparation, base, and preparation–base on FS was statistically significant (*p* < 0.05) (Table 2).

**Figure 5:**
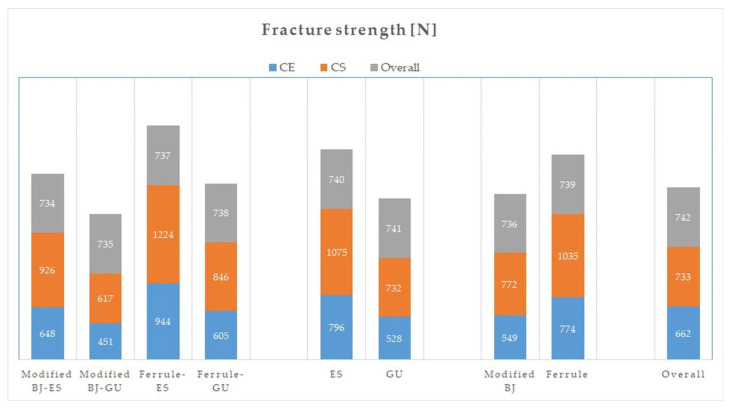
Fracture strength test results. CE, Cerec Blocs; CS, Cerasmart 270; BJ, butt joint; ES, everStick NET; GU, G-aenial Injectable.

When the material was analyzed regardless of preparation and base, the FS obtained for CE was 662 ± 209 N, while for CS it was 903 ± 238 N (*p* < 0.001). When preparation was analyzed regardless of material and base, the FS obtained for the modified BJ was 660 ± 199 N, while for ferrule it was 905 ± 245 N (*p* < 0.001). When base was analyzed regardless of material and preparation, the FS obtained for ES was 935 ± 236 N, while for GU it was 630 ± 163 N (*p* < 0.001) (Table 3 and Figure 5).

The results of the pairwise interactions are shown in Table 4. When material–preparation was analyzed regardless of base, the FS was as follows: CS–ferrule (1035 ± 218 N), CE–ferrule (774 ± 201 N), CS–modified BJ (772 ± 182 N), CE–modified BJ (549 ± 150 N), respectively (*p* > 0.05). When material–base was analyzed regardless of preparation, the FS was as follows: CS-ES (1075 ± 185 N), CE-ES (796 ± 200 N), CS-GU (732 ± 144 N), and CE-GU (528 ± 109 N), respectively (*p* > 0.05). When preparation–base was analyzed regardless of material, the FS was as follows: ferrule–ES (1084 ± 180 N), modified BJ-ES (787± 190 N), ferrule–GU (725 ± 153 N), modified BJ-GU (534 ± 109 N), respectively (*p* = 0.044).

**Figure 6:**
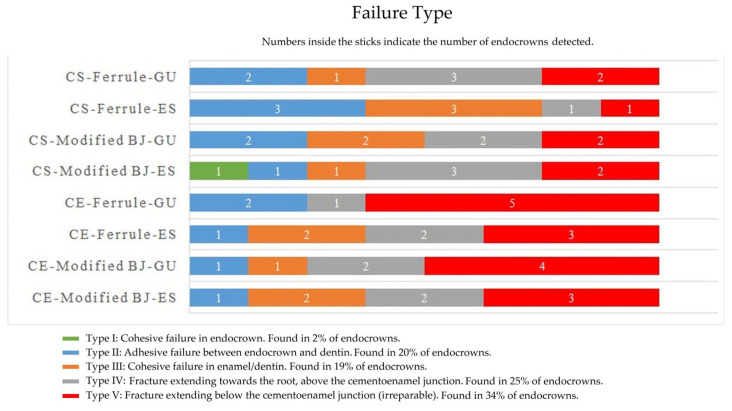
Results for failure types. CE, Cerec; CS, Cerasmart 270; BJ, butt joint; ES, everStick NET; GU, G-aenial Injectable.

The failure types are shown in Figure 6. According to the results, the failure types were Type V (22 teeth; 34%), Type IV (16 teeth; 25%), Type III (12 teeth; 19%), Type II (13 teeth; 20%), and Type I (1 tooth; 2%).

**Figure 7:**
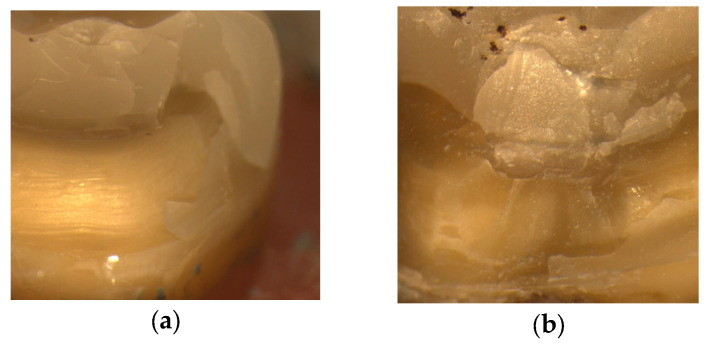

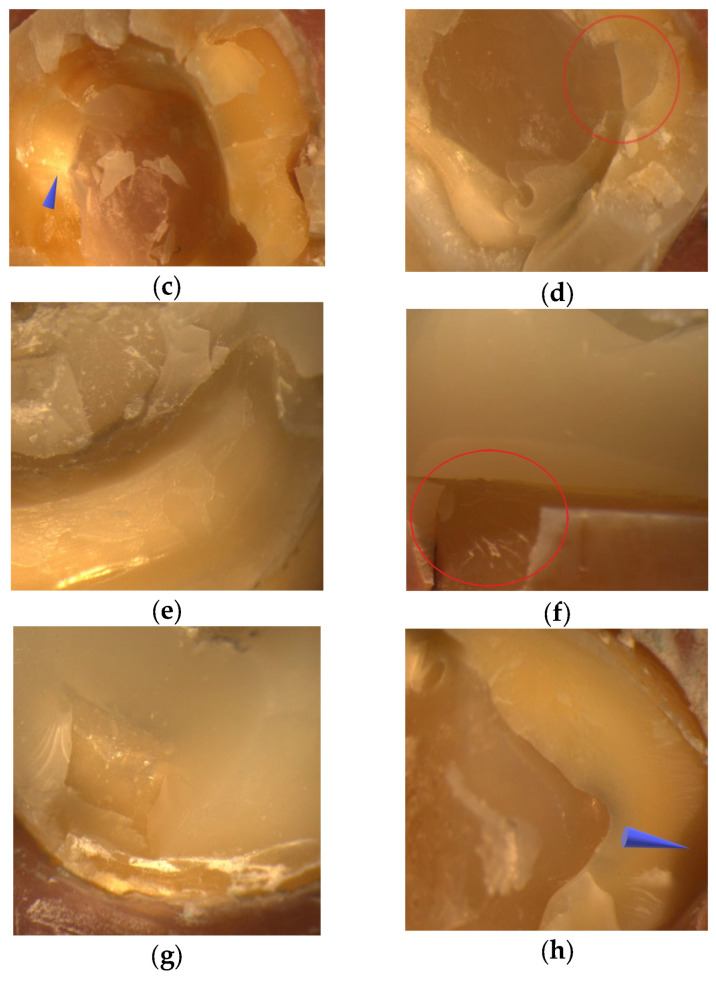
Stereomicroscopic examination after fracture testing. (**a**) Tooth with fiber ribbon base and intact ferrule. (**b**) Tooth with fiber ribbon base and fractured ferrule. (**c**) Tooth with fiber ribbon base and fractured modified butt joint preparation. A fracture line was detected in the blue arrow. (**d**) Tooth with fiber ribbon and retention groove. The connection between the material and the groove was detected in the red circle. (**e**) Hybrid ceramic restoration without a fiber ribbon base and with intact ferrule. (**f**) Hybrid ceramic restoration with fiber ribbon base and modified butt joint preparation. An irreparable fracture was detected in the red circle. (**g**) View of a tooth without fiber ribbon base and with intact ferrule. (**h**) View of a tooth without fiber ribbon base and with fractured modified butt joint preparation. An irreparable fracture was detected in the blue arrow.

Figure 7 shows stereomicroscopic images. In Figure 7a, in a ceramic endocrown with a fiber ribbon base, the ferrule is intact but the bond between the material and dentin is broken. In Figure 7b, in a tooth with a fiber ribbon base and ferrule, the bond between the material and the ceramic is broken and the ferrule is fractured. In Figure 7c, the blue arrow shows a horizontally spreading crack in a tooth with a fiber ribbon base and a modified BJ. In Figure 7d, the red circle shows the relationship between the groove and the base in another tooth with a fiber ribbon base. In Figure 7e, the material is found to be fractured but the ferrule is intact in a hybrid ceramic endocrown without a fiber ribbon base. In Figure 7f, the red circle shows an irreparable fracture in a hybrid ceramic endocrown with a fiber ribbon base and a modified BJ. Figure 7g shows that despite the fracture in the material, the overall integrity is maintained in a hybrid ceramic endocrown without a fiber ribbon base and with ferrule. In Figure 7h, the blue arrow shows an irreparable fracture in the ceramic endocrown without a fiber ribbon base and with a modified BJ.

## 4. Discussion

Endocrown restorations are an important type of restoration because they require less tooth preparation and preserve more of the remaining tooth structure in damaged teeth than post-core restorations. To test the long-term success of endocrowns, this study analyzed marginal and internal fit and resistance to fracture under stress (FS). In addition, the feasibility of using the BJ preparation type instead of the ferrule preparation type in cases where the remaining root dentin is limited was investigated. For this purpose, a new BJ preparation type modified with two grooves was evaluated. The effect of reinforcing the base material with a fiber ribbon and of different types of restorative materials on marginal and internal fit and FS was also investigated.

In this study, the first hypothesis regarding marginal and internal fit was partially rejected. Material significantly affected marginal and internal fit, while preparation and base did not. The second hypothesis regarding FS was also partially rejected. Material, preparation, base, and preparation–base significantly affected FS, while material–preparation, material–base, and material–preparation–base did not. The marginal fit of restorations can be assessed by analyzing the line drawn from the crown margin to the tooth margin (AMD) and the vertical gap between the crown and the tooth margin (MD), while the internal fit can be assessed by analyzing the axial and pulpal fit values. Based on previous studies [22,24,31,32,33], threshold values were chosen as 120 µm for AMD, marginal discrepancy, axial fit, and overall fit, and 160 µm for pulpal fit. According to the results of this study, when material was analyzed regardless of preparation and base, the AMD, MD, pulpal fit, axial fit, and overall fit values obtained for the hybrid ceramic (CS) were statistically significantly higher than the feldspathic ceramic (CE). The MD values of both materials did not exceed the threshold, but the AMD values were higher than the threshold (up to 19 µm for CS and 7 µm for CE). Small differences are reported to be clinically insignificant [31]. Therefore, the values obtained for both materials can be considered clinically appropriate. The pulpal and axial fit values of CS were 151 ± 20 µm and 112 ± 10 µm, respectively, whereas those of CE were 139 ± 29 µm and 106 ± 9 µm.

The effect of preparation–base on AMD was not statistically significant, but the partial eta squared value obtained (0.035) was second only to the partial eta squared value obtained for the material (0.076). All AMD results for preparation–base were slightly above the threshold, with the highest AMD value (138 ± 19 µm for the modified BJ-ES) and the lowest AMD value (127 ± 16 µm for the ferrule–ES) differing by only 9 µm. This result supports the idea that changing the base or the type of preparation does not affect the AMD of endocrowns.

The effect of preparation on MD was not statistically significant, but the partial eta squared value obtained (0.039) was second only to the partial eta squared value obtained for the material (0.1). The MD results obtained for the preparation (107 ± 9 µm for the modified BJ and 111 ± 10 µm for the ferrule) were similar and below the threshold. This result supports the idea that both preparation types produce clinically acceptable axial fit in endocrowns, even when the material or base type is changed.

The effect of material–base on the overall fit was not statistically significant; however, the partial eta squared value obtained (0.036) was second only to the partial eta squared value obtained for the material (0.247). According to the overall fit results of material–base, the values obtained were, in descending order, CS-GU (130 ± 12 µm), CS-ES (129 ± 10 µm), CE-ES (123 ± 6 µm), and CE-GU (117 ± 5 µm). This result supports the idea that CE has a slight advantage over CS in terms of overall fit. In addition, once a material has been selected for endodontic treatment, it can be stated that the base type has a tendency not to affect the overall fit of the restoration made with that material.

In this study, digital measurements were taken using Omnicam (Sirona Dental, Behnsheim, Germany), similar to previous studies [31,34]. In the study by de Paula Siveria et al. [31], the fit results obtained for ceramics were in the order of pulpal > AMD > MD, similar to this study, but the values were found to change when the device was changed. The cement thickness of the restorations can be adjusted to different sizes as far as the CAD software allows. De Fayed et al. [34] reported that the cement spacing should be 80 µm to achieve good FS. Dauti et al. [35] reported that restorations with uniform cement thickness would have better biomechanical behavior. In accordance with the above studies, the pulpal and axial cement spacing in this study was equal at 80 µm.

Although the teeth in this study were prepared according to the basic preparation rules, the presence of undesirable defects such as sharp corners and edges and undercuts may have adversely affected the fit of the restorations. In addition, the restorations placed on the teeth may have moved unnoticed during the triple scan. Svanborg et al. [36], in a study comparing the silicone replica and triple-scan methods, reported that if pressure had been applied during the triple scan, fit values lower than the current values could have been obtained. Another factor that may cause measured values to differ from calculated values has been reported to be the knowledge and skill of the personnel using the CAD/CAM device [24].

Consistent with previous studies [27,37], the pulpal fit values in this study were higher than the axial fit and MD values. The differences in axial and pulpal fit may be due to a phenomenon that causes distortion when the burs capture the pulpoaxial angles [31]. Dahl et al. [38] stated that it would be logical to believe that this is due to the size and shape of the burs, which are incompatible with the internal corners. Taha et al. [4] reported that the more complex the preparation design, the worse the fit between the endocrown and the inner surface of the tooth.

Methods that provide two-dimensional images, such as silicone replica, can be used to assess marginal and internal fit. However, three-dimensional imaging methods, such as microcomputed tomography and the triple-scan method, are becoming increasingly popular. Different intraoral scanners can be used for the triple-scan method. It has been reported that the main factor affecting the measurement quality of intraoral scanners is the complexity of the preparation [39]. In a study, it was found that the depth of the pulp should be between 2 and 5 mm for accurate measurements with intraoral scanners in endocrowns [40]. In this study, the triple-scan method was used because of its good reproducibility, reliability, and ability to provide a comprehensive examination. It has been reported that the more points analyzed, the more accurate the results [41]. Contrepois et al. [42] stated that the total number of measurement points should be at least 18. Therefore, in this study, a method was designed in which a total of 24 measurements were taken on one tooth.

As the abrasiveness of the burs is higher in materials with low hardness and elastic modulus, the risk of discrepancy between tooth and restoration may be increased [24]. The first material used in this study was a feldspathic ceramic with an elastic modulus of 45 GPa and a Vickers hardness of 640 HV. The other material was a hybrid ceramic containing 29% resin matrix and 71% filler with an elastic modulus of 9.6 GPa and a Vickers hardness of 89 HV [43]. These materials were industrially produced and could be placed in the mouth immediately after milling. Furthermore, the milling process is carried out using a software program that takes into account the physical properties of the materials [44]. Probably for this reason, no major differences were found between the materials in terms of marginal and internal fit.

It has been reported that the occlusal force occurring in the molar region was 222–445 N (mean 322.5 N), and this value increased up to 520–800 N (660 N) during tooth clenching. As the habit of clenching is a problem that affects most people today, 660 N was chosen as the threshold in this study [45]. According to the results obtained in this study, CS (903 ± 238 N) had a statistically significantly higher FS than CE (662 ± 209 N) when the material was analyzed regardless of preparation and base; ferrule (905 ± 245 N) had a higher FS than modified BJ (660 ± 199 N) when the preparation was analyzed regardless of material and base; and base with fiber ribbon (ES) (935 ± 236 N) had a higher FS than base without fiber ribbon (GU) (630 ± 163 N) when the base was analyzed regardless of preparation and material. It was found that CE and modified BJ were at the limit of the threshold and GU was below the threshold, while CS, ferrule, and ES were well above the threshold. This result showed that, depending on the type of preparation or restorative material, GU may not be recommended for use in certain configurations.

The effect of material–base on FS was not statistically significant, but it had a partial eta squared value of 0.036. According to the FS results for material–base, the values obtained were, in descending order, CS-ES (1075 ± 185 N), CE-ES (796 ± 200 N), CS-GU (732 ± 144 N), and CE-GU (528 ± 109 N). The threshold was not exceeded by the CE-GU pair. This result supports the idea of using CS with ES or GU and CE with ES for endocrowns with high FS.

When triple interactions were analyzed, FS decreased in order from largest to smallest as follows: CS–ferrule–ES, CE–ferrule–ES, CS–modified BJ–ES, CS–ferrule–GU, CE–modified BJ–ES, CS–modified BJ–GU, CE–ferrule–GU, CE–modified BJ–GU. When the results were interpreted according to the threshold, it was understood that if a modified BJ preparation was to be made, the material should be CS and the base should be ES. If the material was CE, the FS decreased by 278 N but was still close to the threshold. If both material and base were changed, the FS decreased by 475 N and therefore the most vulnerable endocrowns were formed. It was also understood that CS-FR-ES should be the first choice for endocrowns with the highest FS, and when the material is changed, as long as the ferrule and ES are not abandoned, an endocrown with a high FS could still be produced.

In this study, in agreement with previous studies [18,46], not only was the FS of CS higher than that of CE, but the number of repairable failure types was also higher than that of CE. Feldspathic ceramics are more brittle than hybrid ceramics, and the main disadvantage of brittle materials is their low fracture toughness. In the study by Dikici et al. [46] for CS and FC, Type I was the dominant failure type. Although Type I was found in only one tooth in this study, the ratio of repairable to irreparable failure types was almost 2:1. When the elastic modulus of materials is similar to that of dentin, their flexible behavior and energy dissipation properties are enhanced [47]. Compared to CE, the elastic modulus of CS is closer to dentin. Furthermore, the higher flexural strength of CS compared to CE (246 MPa for CS and 113 MPa for CE) is another factor that provides better FS [18,46]. In general, ceramics fracture according to Griffith’s theory, which explains the propagation of internal cracks and the fracture initiation mechanism [28]. CS is more in accordance with the Dugdale cracking theory, as it contains resin matrix and the first cracks formed give the mass the beginning of plasticity. As the load increases, plasticity then spreads and crack resistance increases along the long crack [48].

On the other hand, in a study evaluating endocrowns with CS, Zheng et al. [47] reported that keeping the crown margin high would increase FS. However, healthy tissue may not be available to keep the crown margin elevated at all times. Ghoul et al. [30] analyzed a BJ design with two grooves, similar to the present study, but horizontal and axial forces were applied instead of oblique forces, and the grooves were reported to increase FS. As similar results were obtained in the present study, it appears that the preparation of a BJ with two grooves may be an alternative to keeping the crown margin high.

In this study, a portion of the flowable resin base was modified with a fiber ribbon. This ribbon consists of silanized, bidirectional glass fibers and a resin matrix containing bisphenol A-glycidyl methacrylate (Bis-GMA) and polymethyl methacrylate (PMMA). The material is in the form of a 0.05 mm thick sheet and is applied by cutting [14]. In this study, in agreement with previous studies [49,50] in which the base was reinforced with fiber ribbon, FS was found to be increased in restorations with fiber ribbon, and the number of teeth with irreparable failure types was reduced.

In this study, fiber ribbon consisting of bidirectional glass fiber ribbons was cut, arranged, and embedded in two layers of flowable resin composite. Another study [50] using a similar technique and a different brand of fiber ribbon (Ribbond; Ribbond Inc, Seattle, WA, USA) found, similar to this study, a decrease in the rate of irreparable fractures and an increase in Type III fractures.

Similar to this study, Anton Y Otero et al. [15] used the same hybrid ceramic material and two different base materials. One was a flowable resin composite (Everx Flow; GC Corp, Tokyo, Japan) containing multidirectional glass fibers, and the other was everStick NET. EverX Flow contains Bis-GMA, triethylene glycol dimethacrylate (TEGDMA), PMMA, silanated short glass fibers, and barium glass fillers. The study reported that the base with three layers of fiber ribbons increased the FS, which was made possible by the bidirectional fiber ribbon structure of everStick NET, and the fiber material also served to deflect the crack direction [15].

The improving effect on the FS and failure type of the fiber ribbon can be explained by the Krenchel factor. Since ES has bidirectional fiber orientation (Krenchel factor: 0.5), the efficiency of the fibers is high [51]. The position of the fiber ribbon in the composite layer can also influence the FS. Unwanted defects that occur during the correction of the base can cause the ribbon to be carried upwards, and as a result the FS can be positively or negatively affected [52]. In this study, the distance between the margin of the crown and the base surface was regularly checked with a periodontal probe for standardization.

In this study, the ferrule statistically significantly increased the FS of the materials compared to the modified BJ. Einhorn et al. [5], similar to this study, found higher FS in teeth with ferrules compared to teeth with BJs, but a 1 mm height for ferrules was more favorable than 2 mm. Abduljawad and Rayyan [53] reported that 2 mm ferrules resulted in limited dentin tissue at the preparation margin. If there is weak dentin at the root, the possibility of fracture propagation to the root is increased. Therefore, when designing a ferrule preparation, care should be taken to maintain a 2 mm band of intact dentin [5]. In this study, when the results were analyzed regardless of the material, statistically significantly higher FS was found in teeth with an ES base and a modified BJ compared to teeth with a GU base and ferrule. This result indicates that modified BJs are preferred in cases that prevent ferrule application, such as insufficient remaining intact dentin tissue, if a base with fiber ribbon is prepared.

This study presented various treatment alternatives, including ferrule, modified BJs, bases with and without fiber ribbon, ceramic, and hybrid ceramic. Post-core restoration requires more tissue removal than endocrowns and does not allow the tooth to be restored in the event of failure, which is why the use of endocrowns has increased in recent years. This study investigating the biomechanical performance of endocrowns is therefore of significant clinical importance. In addition, the investigation of types of preparation, materials, and base, as well as the presentation of new alternatives, further enhances the clinical significance of this study. The effect of cementation on marginal and internal fit and FS was not analyzed in this study because it was not desirable to extend the study to a dimension that would deviate from its focus, including the fit assessment. Furthermore, it may also be necessary to evaluate materials with different manufacturing and preparation methods. Another limitation was that the effect of intraoral conditions on the internal and marginal fit of restorations was not investigated. Future studies are needed to investigate the effect of different restorative materials and types of base and finish lines on the marginal and internal fit and FS of endocrowns.

## 5. Conclusions

The findings within the constraints of this study are summarized as follows:

**Material Comparison:** When analyzed irrespective of preparation and base, hybrid ceramics demonstrated higher values in absolute marginal discrepancy (AMD), marginal discrepancy (MD), and axial, pulpal, and overall fit compared to ceramic restorations. Additionally, the fracture strength of teeth restored with hybrid ceramics was superior to those restored with ceramics.

**Preparation Comparison:** When the preparation type was considered independently of base and material, teeth with ferrules and modified butt joints exhibited similar AMD, MD, and axial, pulpal, and overall fit values. However, the fracture strength was significantly higher in teeth with ferrules compared to those with modified butt joints.

**Base Comparison**: In the analysis where base was considered independently of preparation and material, the AMD, MD, and axial and overall fit values were comparable between teeth with and without fiber bases. However, teeth with fiber bases showed slightly superior pulpal fit. Moreover, the fracture strength was significantly higher in teeth with fiber bases than in those without.

**Fit Value Ranking**: The fit values, ranked from highest to lowest, were as follows: pulpal, AMD, overall, MD, and axial. Hybrid ceramic restorations with ferrules and fiber bases generally exhibited the highest fracture strength, whereas ceramic restorations without fiber bases and modified butt joints exhibited the lowest fracture strength.

## Figures and Tables

**Table 1:** Materials used in the study.

Intended Use	Code	Name	Feature	Content (% in Weight)	Manufacturer
Pulpal base	GU	G-ænial Universal Injectable	Light-cured injectable composite	31% matrix (methacrylate monomer), 69% fillers (silica, barium glass)	GC Corp, Tokyo, Japan
	ES	everStick NET	Fiber ribbon	Bidirectional glass fibers, polymer/resin gel matrix (PMMA, Bis-GMA)	GC Corp, Tokyo, Japan
Restorative material	CE	Cerec Blocs	Feldspathic ceramic blocs	56–64% SiO_2_, 20–23% Al_2_O_3_, 6–9% Na_2_O, 6–8% K_2_O, 0.3–0.6% other oxides	Sirona Dental, Bensheim, Germany
	CS	Cerasmart 270	Hybrid ceramic blocs	29% matrix (UDMA, Bis-MEPP, DMA), 71% fillers (300 nm barium glass; 20 nm SiO_2_ nanoparticles)	GC Corp, Tokyo, Japan

SiO_2_, silicium dioxide; Al_2_O_3_, aluminum oxide; Na_2_O; sodium oxide; K_2_O, potassium oxide; Bis-GMA, bisphenol A-glycidyl methacrylate; PMMA, Polymethyl methacrylate; UDMA, urethane dimethacrylate; Bis-MEPP, Bismethacryloxyethoxydiphenylpropane; DMA, dodecyl dimethacrylate.

**Table 2:** ANOVA results for AMD, MD, axial, pulpal, and overall fits, and fracture strength.

	Source	Sum of Squares	df	F	*p*-Value	Partial Eta Squared
**AMD**	Material	2626.563	1	4.596	0.036	0.076
	Preparation	156.25	1	0.273	0.603	0.005
	Base	12.25	1	0.021	0.884	0.001
	Material–preparation	27.563	1	0.048	0.827	0.001
	Material–base	612.563	1	1.072	0.305	0.019
	Preparation–base	1156	1	2.023	0.16	0.035
	Material–preparation–base	248.063	1	0.434	0.513	0.008
**MD**	Material	756.25	1	5.589	0.022	0.091
	Preparation	34.516	1	0.255	0.616	0.005
	Base	54.391	1	0.402	0.529	0.007
	Material–preparation	2.641	1	0.02	0.889	0.001
	Material–base	102.516	1	0.758	0.388	0.013
	Preparation–base	60.063	1	0.444	0.508	0.008
	Material–preparation–base	18.063	1	0.133	0.716	0.002
**Axial fit**	Material	650.25	1	6.197	0.016	0.1
	Preparation	240.25	1	2.29	0.136	0.039
	Base	22.563	1	0.215	0.645	0.004
	Material–preparation	6.25	1	0.06	0.808	0.001
	Material–base	52.563	1	0.501	0.482	0.009
	Preparation–base	39.063	1	0.372	0.544	0.007
	Material–preparation–base	5.063	1	0.048	0.827	0.001
**Pulpal fit**	Material	2588.266	1	13.491	<0.001	0.094
	Preparation	1113.891	1	0.281	0.598	0.005
	Base	3150.016	1	0.281	0.598	0.005
	Material–preparation	446.266	1	0.052	0.821	0.001
	Material–base	293.266	1	0.206	0.651	0.004
	Preparation–base	43.891	1	0.116	0.735	0.002
	Material–preparation–base	54.391	1	0.323	0.572	0.006
**Overall fit**	Material	1602.501	1	18.398	<0.001	0.247
	Preparation	78.766	1	0.904	0.346	0.016
	Base	126.563	1	1.453	0.233	0.025
	Material–preparation	6.25	1	0.072	0.79	0.001
	Material–base	183.941	1	2.112	0.152	0.036
	Preparation–base	153.915	1	1.767	0.189	0.031
	Material–preparation–base	1.806	1	0.021	0.886	0.001
**Fracture strength**	Material	932,575.214	1	87.424	<0.001	0.61
	Preparation	955,052.36	1	89.531	<0.001	0.615
	Base	1,493,630.413	1	140.02	<0.001	0.714
	Material–preparation	5878.626	1	0.551	0.461	0.01
	Material–base	22,564.026	1	2.115	0.151	0.036
	Preparation–base	45,216.506	1	4.239	0.044	0.07
	Material–preparation–base	5402.61	1	0.506	0.48	0.009

Significant associations (*p* < 0.05) indicated in bold. AMD, absolute marginal discrepancy; MD, marginal discrepancy.

**Table 3:** Means ± standard deviations of AMD, MD, axial, pulpal, and overall fits, and fracture strength.

Material	Preparation	N	Base	Means ± Standard Deviations					
				AMD (µm)	MD (µm)	Axial (µm)	Pulpal (µm)	Overall (µm)	FS (N)
**CE**	Modified BJ	8	ES	136 ± 18	112 ± 7	106 ± 9	146 ± 22	124 ± 6	648 ± 148
		8	GU	119 ± 15	108 ± 8	102 ± 7	128 ± 30	114 ± 3	451 ± 67
		16	Total	128 ± 18	110 ± 7	104 ± 8	137 ± 27	119 ± 7	549 ± 150
	Ferrule	8	ES	122 ± 25	107 ± 10	109 ± 13	149 ± 31	122 ± 6	944 ± 117
		8	GU	129 ± 32	109 ± 11	107 ± 6	131 ± 33	119 ± 6	605 ± 86
		16	Total	125 ± 28	108 ± 10	108 ± 10	140 ± 32	120 ± 6	774 ± 201
	Total	16	ES	129 ± 22	109 ± 9	107 ± 11	148 ± 26	123 ± 6	796 ± 200
		16	GU	124 ± 25	108 ± 9	104 ± 7	129 ± 30	117 ± 5	528 ± 109
		32	Total	127 ± 23	108 ± 9	106 ± 9	139 ± 29	120 ± 6	662 ± 209
**CS**	Modified BJ	8	ES	140 ± 22	115 ± 8	111 ± 11	151 ± 23	129 ± 13	926 ± 106
		8	GU	143 ± 18	118 ± 11	110 ± 8	138 ± 20	127 ± 11	617 ± 72
		16	Total	142 ± 19	116 ± 10	111 ± 10	145 ± 22	128 ± 11	772 ± 182
	Ferrule	8	ES	131 ± 27	113 ± 16	112 ± 11	161 ± 13	129 ± 8	1224 ± 105
		8	GU	143 ± 25	118 ± 15	115 ± 11	155 ± 17	133 ± 12	846 ± 98
		16	Total	137 ± 26	115 ± 15	114 ± 10	158 ± 15	131 ± 10	1035 ± 218
	Total	16	ES	136 ± 24	114 ± 12	112 ± 11	156 ± 19	129 ± 10	1075 ± 185
		16	GU	143 ± 21	118 ± 13	113 ± 10	147 ± 20	130 ± 12	732 ± 144
		32	Total	139 ± 23	116 ± 13	112 ± 10	151 ± 20	130 ± 11	903 ± 238
**Overall**	Modified BJ	16	ES	138 ± 19	114 ± 7	108 ± 10	149 ± 19	127 ± 10	787 ± 190
		16	GU	131 ± 20	113 ± 11	106 ± 8	133 ± 25	121 ± 10	534 ± 109
		32	Total	135 ± 20	113 ± 9	107 ± 9	141 ± 25	124 ± 10	660 ± 199
	Ferrule	16	ES	127 ± 16	110 ± 13	110 ± 12	155 ± 24	126 ± 8	1084 ± 180
		16	GU	136 ± 29	113 ± 14	112 ± 9	143 ± 28	126 ± 12	725 ± 153
		32	Total	131 ± 27	112 ± 13	111 ± 10	149 ± 26	126 ± 10	905 ± 245
	Total	32	ES	133 ± 23	111 ± 11	110 ± 11	152 ± 23	126 ± 9	935 ± 236
		32	GU	134 ± 25	113 ± 12	109 ± 9	138 ± 27	123 ± 11	630 ± 163
		64	Total	133 ± 24	112 ± 11	109 ± 10	145 ± 26	125 ± 10	783 ± 253

CE, Cerec; CS, Cerasmart 270; ES, everStick NET; GU, G-aenial Injectable; BJ, butt joint; AMD, absolute marginal discrepancy; MD, marginal discrepancy; FS, fracture strength.

**Table 4:** Pairwise interaction results for fracture strength (N).

	**Material–Preparation**			
		**Mean**	**95% Confidence Interval**	
			**Lower Bound**	**Upper Bound**
**CE**	Modified BJ	549	497	601
	Ferrule	774	722	826
**CS**	Modified BJ	771	719	823
	Ferrule	1035	98	1086
	Material–base			
		Mean	95% Confidence interval	
			Lower bound	Upper bound
**CE**	ES	795	744	847
	GU	527	476	579
**CS**	ES	1074	102	1126
	GU	731	680	783
	Preparation–base			
		Mean	95% Confidence interval	
			Lower bound	Upper bound
**Modified BJ**	ES	786	734	838
	GU	534	482	586
**Ferrule**	ES	1084	1032	1135
	GU	725	673	777

CE, Cerec; CS, Cerasmart 270; BJ, butt joint; ES, everStick NET; GU, G-aenial Injectable.

## Data Availability

Data are contained within the article.

## Abbreviations

The following abbreviations are used in this manuscript:

FS	Fracture strength
BJ	Butt joint
CEJ	Cementoenamel junction
LD	Linear dichroism
MD	Marginal discrepancy
AMD	Absolute marginal discrepancy
CAD/CAM	Computer-aided design/computer-aided manufacturing
CE	Cerec Blocs
CS	Cerasmart 270
N	Newton
ANOVA	Analysis of variance
Bis-GMA	Bisphenol A-glycidyl methacrylate
PMMA	Polymethyl methacrylate
TEGDMA	Triethylene glycol dimethacrylate
